# Retroperitoneal Compared to Transperitoneal Approach for Open Abdominal Aortic Aneurysm Repair Is Associated with Reduced Systemic Inflammation and Postoperative Morbidity

**DOI:** 10.1055/s-0042-1749173

**Published:** 2022-12-20

**Authors:** Damian M. Bailey, George A. Rose, Daniel O'Donovan, Dafydd Locker, Ian R. Appadurai, Richard G. Davies, Richard J. Whiston, Mohamad Bashir, Michael H. Lewis, Ian M. Williams

**Affiliations:** 1 Neurovascular Research Laboratory, Faculty of Life Sciences and Education, University of South Wales, Pontypridd, United Kingdom; 2 Department of Anaesthetics, University Hospital of Wales, Cardiff, United Kingdom; 3 Department of Vascular Surgery, University Hospital of Wales, Cardiff, United Kingdom

**Keywords:** abdominal aortic aneurysm repair, retroperitoneal, transperitoneal, cardiorespiratory fitness, inflammation

## Abstract

**Background** In the United Kingdom, the most common surgical approach for repair of open abdominal aortic aneurysms (AAAs) is transperitoneal (TP). However, retroperitoneal (RP) approach is favored in those with more complex vascular anatomy often requiring a cross-clamp on the aorta superior to the renal arteries. This study compared these approaches in patients matched on all major demographic, comorbid, anatomic, and physiological variables.

**Methods** Fifty-seven patients (TP: *n* = 24; RP: *n* = 33) unsuitable for endovascular aneurysm repair underwent preoperative cardiopulmonary exercise testing prior to open AAA repair. The surgical approach undertaken was dictated by individual surgeon preference. Postoperative mortality, complications, and length of hospital stay (LoS) were recorded. Patients were further stratified according to infrarenal (IR) or suprarenal/supraceliac (SR/SC) surgical clamping. Systemic inflammation (C-reactive protein) and renal function (serum creatinine and estimated glomerular filtration rate) were recorded.

**Results** Twenty-three (96%) of TP patients only required an IR clamp compared with 12 (36%) in the RP group. Postoperative systemic inflammation was lower in RP patients ( *p* = 0.002 vs. TP) and fewer reported pulmonary/gastrointestinal complications whereas renal impairment was more marked in those receiving SR/SC clamps ( *p* < 0.001 vs. IR clamp). RP patients were defined by lower LoS ( *p* = 0.001), while mid-/long-term mortality was low/comparable with TP, resulting in considerable cost savings.

**Conclusion** Despite the demands of more complicated vascular anatomy, the clinical and economic benefits highlighted by these findings justify the more routine adoption of the RP approach for complex AAA repair.

## Introduction

Open surgical repair of an abdominal aortic aneurysm (AAA) incurs significant physiological stress and morbidity. In a recent systematic review of 21 case series comprising 1,575 patients, 30-day or in-hospital mortality after open juxtarenal AAA (JRAAA) repair was 4.1%. 1 In a contemporary series of patients included in the Vascular Study Group of New England registry, perioperative mortality was 3.6% in 443 patients after elective open surgical repair for a JRAAA or pararenal AAA. 2 Morbidity is significantly higher than mortality, with an estimated prevalence of 28% following open repair and 12% following endovascular repair (EVAR). 3 A robust risk assessment is therefore necessary to identify high-risk patients preoperatively, assess comorbidities, and plan safe intervention.

In conjunction with classical cardiovascular risk factors, preoperative cardiopulmonary exercise testing (CPET) constitutes part of the integrated risk assessment to establish if a patient has adequate cardiorespiratory fitness (CRF) to undergo major aortic surgery. 4 5 CPET has gained popularity as a routine diagnostic test in the United Kingdom 6 with reports of up to 84% of AAA patients undergoing CPET preoperatively. 7 However, uptake of CPET as a preoperative assessment tool outside the United Kingdom is unknown. The predictive value of CRF for mid- and long-term survival in patients undergoing elective open AAA repair appears well established, 8 9 10 including its ability to forecast postoperative morbidity, 10 11 12 thus supporting its role in guiding perioperative care.

In the United Kingdom the most common surgical approach for open AAA repair is transperitoneal (TP) which provides excellent access to the infrarenal (IR) aorta. The technically more complex retroperitoneal (RP) approach confers the benefit of avoiding breaching the peritoneal cavity associated with a traditional midline incision. The RP approach also offers many advantages in those with a short IR aortic neck by enabling exposure of the lateral wall of the aorta proximally as far as the origin of the celiac axis. However, suprarenal aortic cross-clamping increases the risk of developing renal ischemia-reperfusion injury. 13 Yet, to what extent the surgical approach impacts patient outcome remains to be established.

To address this, we conducted a nonrandomized retrospective study to compare how these separate surgical approaches to open AAA repair influence postoperative morbidity and mortality, exploring also the potentially differential, underlying mechanisms, and associated hospital costs. Only those patients turned down for EVAR were included. Furthermore, there were no differences in baseline demographic and comorbid variables between the TP and RP groups. We hypothesized that despite the demands of more complicated baseline vascular anatomy, the RP approach would be associated with lower postoperative morbidity thanks to a lower incidence of gastrointestinal complications and corresponding suppression of the systemic inflammatory response reflected by consistently lower C-reactive protein (CRP) levels.

## Methods

### Ethics

The study was approved by the Cardiff and Vale University Health Board (#15/AIC/6352). Patient consent was waived given that the study constituted a retrospective analysis of an anonymized database.

### Design

This was a nonrandomized, cross-sectional retrospective analysis of patients who had undergone open surgical AAA repair performed by either TP ( *n* = 24) or RP ( *n* = 33) approaches without inclusion/exclusion criteria applied prospectively.

### Patients

All patients included in the study were scheduled for elective surgery over a 7-year period from 2009 to 2016 and were followed up for 2 years. We specifically selected this period since all data sets (including accompanying CPET metrics) were available for analysis on a centralized database. Patients were drawn from a single center (Department of Surgery, University Hospital of Wales, Wales, U.K.) anonymized National Health Service database of consecutive patients who had undergone preoperative CPET for elective open AAA repair, having initially been turned down for EVAR for anatomical reasons only. Patients were identified following referral based on the ultrasound detection of an aneurysm via the Wales AAA Screening Program and/or following an incidental finding on computed tomography (CT, see below). For open surgical intervention to be considered, the minimal diameter in the anterior-posterior or transverse planes was 50 mm for women and 55 mm for men. 14 15 Those patients deemed to have a short IR neck and who may require a suprarenal (SR) cross-clamp underwent surgery via a RP approach. Each case was discussed at a multidisciplinary meeting and the operative approach ultimately decided by consultant surgeons in a nonrandomized manner.

### Surgical Approach

Both the TP and RP approach were performed under general anesthesia with an epidural spinal catheter and radial artery monitoring of blood pressure. All had a urinary catheter, and patients were postoperatively monitored in intensive care or high dependency units (ICU/HDU) for at least 24 hours. Nasogastric tubes were not routinely inserted, and resumption of oral fluids and diet was encouraged within 24 to 48 hours following surgery. The TP approach was performed via a standard midline incision, giving excellent access to the IR aorta and iliac arteries. For those undergoing the RP approach and requiring a cross-clamp proximal to the renal arteries, the incision was extended laterally into the 8th/9th rib space. This extension of the incision provides an 8- to 10-cm extra access without breaching the pleura and provides excellent exposure of the proximal abdominal aorta. However, there is little to no morbidity associated with entry into the pleura and all patients underwent chest X-ray postprocedure with none requiring a drain. If on preoperative imaging the aortic clamp was planned to be placed infrarenally then an incision was positioned on the 10th to 11th ribs.

### Measurements

#### Demographics

Patient data including sex, age, body mass index, clinical history, and all metabolic data (see below) were collected via the Cardiff and Vale University Health Board Clinical Portal and following examination of clinical notes.

#### Computed Tomography

All patients prior to operative intervention underwent thin-slice contrast-enhanced arterial-phase CT angiography on a 64-slice multidetector scanner (Optima CT660, GE Healthcare, Little Chalfont, United Kingdom). The maximum (inner to inner wall) diameter in the anteroposterior plane was recorded as well as tortuosity and diameter of the iliac arteries. The origin of the renal arteries was assessed in relation to the commencement of the aneurysm. The diameter and any tortuosity or calcification of the aortic neck was also documented particularly if an endovascular stent graft was being considered. 16 The intra- and intercoefficients of variation (CVs) were < 5%.

#### Spirometry and Cardiopulmonary Exercise Testing

Preoperative resting spirometry (flow-volume loops) and CPET were performed and reported by one of two consultant anesthetists (R.G.D./I.R.A.) in accordance with the American Thoracic Society and American College of Physicians guidelines. 17 An exercise protocol was employed whereby patients cycled at 60 revolutions per minute for 3 minutes in an unloaded freewheeling state followed by a progressively ramped period of exercise (5–15 W/min based on mass, stature, age, and sex) to volitional or symptom-limited termination, followed by 3-minute recovery. 18 Medgraphics Breeze software automatically determined peak oxygen uptake (  , defined as the highest  during the final 30 seconds of exercise reported), oxygen uptake efficiency slope, 19 and peak O _2_ pulse (O _2_ pulse). The anaerobic threshold (AT) was manually interpreted using the V-slope method 20 supported by comparison of end-tidal O _2_ tension (ETO _2_ ) and ventilatory equivalent for O _2_ (  ) plots. The ventilatory equivalent for carbon dioxide (  ) was identified at the AT. Unfit/fit stratification was determined by peak oxygen uptake values </> 16 mL O _2_ /kg/min respectively according to the European Association for Cardiovascular Prevention and Rehabilitation/American Heart Association Scientific Statement. 21

#### Blood Sampling

Blood samples were obtained by routine venipuncture and assayed for hemoglobin (Hb). Remaining samples were centrifuged at 600 × *g* (4°C) for 10 minutes prior to immediate analysis for serum creatinine and CRP. Samples were obtained 2 ± 4 days prior to surgery, immediately after surgery, and, at 2 and 14 days (creatinine) or 1, 3, and 5 days (CRP) following surgery.

*Hb* : total Hb was determined using an ABL800 FLEX blood gas analyzer (Radiometer UK Limited, Crawley, UK). The intra- and inter-CVs were < 5%.

*Serum creatinine* : serum creatinine was analyzed enzymatically on a Cobas 6000 analyzer (Roche Diagnostics, Indianapolis, IN). The intra- and inter-CVs were < 5%.

*Estimated glomerular filtration rate (eGFR)* : this was calculated indirectly using the simplified Modification of Diet in Renal Disease equation 22 given by:

eGFR = 186 × serum creatinine ^−1.154^ × age ^−0.203^ [× 0.742 (if patient is female)]

*CRP* : (high sensitivity) CRP was analyzed using a turbidimetric immunoprecipitation assay on an Abbot Aeroset c8000 analyzer. The intra- and inter-CVs were < 5%.

#### Clinical Outcomes

*Morbidity* : this was measured using the Postoperative Morbidity Survey (POMS) on day 5 postsurgery, an established 9-domain system previously validated in patients undergoing AAA repair, 23 with a specific focus on pulmonary, infectious, renal, gastrointestinal, cardiovascular, neurological, wound, hematological, and pain complications. A score between 0 and 9 was recorded, with clinically significant postoperative morbidity defined as a POMS score ≥ 1 point. Data were also collected for hospital length of stay (LoS), further differentiating between time spent in the ICU and HDU. Patients were discharged from the ICU and HDU after extubation and with the ability to self-ventilate. Oral intake of fluids was encouraged as soon as possible after surgery, with light diet reintroduced usually within 48 hours. All patients were under the care of intensive care doctors, and discharge to the general hospital ward determined by them.

*Mortality* : this was determined by review of Office for National Statistics records and included cause of death, with survival calculated by comparison of surgery and follow-up dates (up to 2 years postoperatively).

#### Economic Evaluation

Average costs associated with surgery, and LoS including time spent on the ward/ICU/HDU, were calculated for each patient based on an algorithm provided by the Welsh Assembly Government for Cardiff & Vale University Health Board within the relevant timeframe.

#### Statistics

Statistical analyses were undertaken using SPSS for Windows (Version 28; IBM, Armonk, NY). Continuous variables are reported as the mean ± standard deviation or median and interquartile range, depending on distribution. Categorical variables are reported as frequencies with percentages. Distribution normality was assessed using Shapiro–Wilk *W* tests. Categorical comparisons were conducted using Chi-square tests or Fisher's exact tests where cell counts were insufficient (any expected frequencies < 5). Differences in renal function (creatinine/eGFR) and systemic inflammatory response (CRP) over time or according to clamp position were analyzed using a two-way (group: TP vs. RP × time: pre- vs. postoperative/Clamp: IR vs. SR + supraceliac [SC]) repeated-measures analysis of variance. Following an interaction, post hoc analysis was performed using a combination of paired (within-group) and independent (between-group) samples *t* -tests. Significance for all two-tailed tests was established at *p* < 0.05.

## Results

### Demographics

Patient demographics including baseline anthropometrics, metabolic function, cardiovascular risk factors, and medications are summarized in Table 1 . With the exception of more female patients in the TP group, both groups were well-matched at baseline prior to surgery.

**Table 1:** Demographics

	Transperitoneal ( *n* = 24)		Retroperitoneal ( *n* = 33)		*p* -Value
*Physiology/biochemistry:*					
Age (y)	71 ± 8		71 ± 6		0.968
Male:female	17:7		32:1		0.007
IMD quintile [median (IQR)]	2 (1–4)		3 (1–5)		0.512
BMI (kg/m ^2^ )	27.7 ± 3.6		27.7 ± 3.5		1.000
Hb (g/dL)	14.4 ± 1.0		14.0 ± 1.1		0.123
Creatinine (μmol/L)	95 ± 13		93 ± 15		0.654
eGFR (mL/min/1.73 m ^2^ )	68 ± 13		75 ± 14		0.054
*Comorbidities:*					
Current smoker [ *n* (%)]	12	(50)	23	(70)	0.132
Past smoker [ *n* (%)]	21	(88)	30	(91)	0.689
IHD [ *n* (%)]	6	(25)	5	(15)	0.499
Hypertension [ *n* (%)]	16	(57)	15	(45)	0.516
Diabetes [ *n* (%)]	1	(4)	1	(3)	1.000
COPD [ *n* (%)]	2	(8)	2	(6)	1.000
*Medication:*					
Aspirin [ *n* (%)]	11	(46)	22	(67)	0.116
Warfarin [ *n* (%)]	1	(4)	1	(3)	1.000
Clopidogrel [ *n* (%)]	2	(8)	0	(0)	0.173
α/β-blockers [ *n* (%)]	1	(4)	1	(3)	1.000
ACEi [ *n* (%)]	6	(25)	16	(48)	0.072
Statins [ *n* (%)]	15	(63)	28	(85)	0.053
Calcium channel antagonists [ *n* (%)]	1	(4)	1	(3)	1.000
*Surgical factors:*					
Aneurysm size (cm)	6.0 ± 0.6		6.2 ± 0.7		0.328
IR clamp [ *n* (%)]	23	(96)	21	(64)	0.004
SR clamp [ *n* (%)]	1	(4)	10	(30)	0.017
SC clamp [ *n* (%)]	0	(0)	2	(6)	0.504

Abbreviations: ACEi, angiotensin-converting enzyme inhibitors; BMI, body mass index; COPD, chronic obstructive pulmonary disease; eGFR, estimated glomerular filtration rate; Hb, hemoglobin; IHD, ischemic heart disease; IMD, index of multiple deprivation scores adjusted to Lower Super Output Areas; IQR, interquartile range; IR, infrarenal; SC, supraceliac; SR, suprarenal.

Note: Values are mean ± standard deviation (SD) or number/percent. *p* -Values obtained using independent samples *t* -tests or Mann–Whitney *U* -tests, Chi-square tests, or Fisher's exact tests where cell counts were insufficient.

### Preoperative Spirometry and CPET

No differences were observed between groups in any of the preoperative lung function or CPET metrics, with approximately 50% of the patients in each group classified as unfit ( Table 2 ).

**Table 2:** Cardiopulmonary data

	Transperitoneal ( *n* = 24)		Retroperitoneal ( *n* = 33)		*p* -Value
*Rest:*					
FVC (L/min)	3.25 ± 1.07		3.50 ± 0.66		0.326
FEV _1_ /FVC (%)	72 ± 9		69 ± 10		0.233
*Submaximal exercise:*					
(mL/kg/min)	11.4 ± 2.3		10.8 ± 2.6		0.425
	31.1 ± 5.3		31.0 ± 4.7		0.899
	32.0 ± 4.2		33.2 ± 5.2		0.335
O _2_ pulse _-AT_ (mL/beat)	9.4 ± 2.3		9.1 ± 2.3		0.609
Workload _-AT_ (W)	58 ± 19		57 ± 17		0.813
OUES [ ][ ]	1,668 ± 565		1,633 ± 434		0.792
*Peak exercise:*					
(mL/kg/min)	15.4 ± 3.7		15.7 ± 3.6		0.762
(% predicted))	66 ± 14		65 ± 18		0.827
RER _PEAK_ (AU)	1.15 ± 0.14		1.12 ± 0.14		0.369
O _2_ pulse _PEAK_ (mL/beat)	11.2 ± 3.0		11.3 ± 3.1		0.892
Workload _Peak_ (W)	95 ± 35		95 ± 28		0.988
Duration _PEAK_ (s)	528 ± 117		541 ± 121		0.680
*Fitness stratification:*					
Unfit [ *n* (%)]	12	(50)	17	(52)	0.910
Fit [ *n* (%)]	12	(50)	16	(48)	0.910

Abbreviations: AT, anaerobic threshold; AU, arbitrary units; FEV _1_ , forced expiratory volume in 1 second; FVC, forced vital capacity; OUES, oxygen uptake efficiency slope; RER, respiratory exchange ratio;  , oxygen uptake;  , ventilatory equivalents for oxygen/carbon dioxide.

Note: Values are mean ± standard deviation (SD) or number (%). Unfit/fit stratification was determined by peak oxygen uptake values </> 16 mL O _2_ /kg/min respectively according to the European Association for Cardiovascular Prevention and Rehabilitation/American Heart Association Scientific Statement. 21 *p* -Values obtained using independent samples *t* -tests and Chi-square tests.

### Surgery

Aneurysm diameters were identical in both groups ( Table 1 ). In the RP group, 30/33 patients (91%) underwent a straight tube graft as operative repair, with the remaining 3/33 patients (9%) undergoing a bifurcation graft to the iliac arteries. Similarly in the TP group, 21/24 patients (88%) received a tube graft while 3/24 patients (12%) required extension to the iliac ( *n* = 2) and femoral arteries ( *n* = 1). In all cases the inferior mesenteric artery was ligated to facilitate aortic exposure, and none were reimplanted following open repair for either approach. One patient in the TP group developed signs of colonic ischemia postoperatively, whereas none were reported in the RP group.

### Postoperative Morbidity

**Fig. 1:**
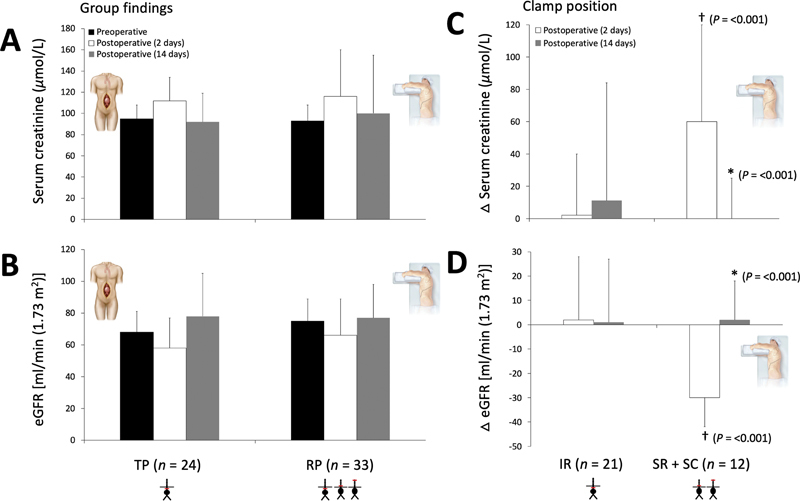
Renal function following transperitoneal (TP) and retroperitoneal (RP) abdominal aortic aneurysm repair. Values are mean ± standard deviation (SD). Group changes ( **A** , **B** ) further differentiated according to clamp position in RP patients ( **C** , **D** ). Δ ( **C** , **D** ), reflects difference relative to preoperative values. eGFR, estimated glomerular filtration rate; IR, infrarenal; SR, suprarenal; SC, supraceliac. ( **A** ) Please note clamp position(s) and see “Methods” (Surgical Approach) for clarification. Group effect: *p* = 0.576; time effect: *p* = 0.001; group × time: *p* = 0.680. ( **B** ) Group effect: *p* = 0.263; time effect: *p* = < 0.001; group × time: *p* = 0.336. ( **C** ) Group effect: *p* = 0.172; time effect: *p* = < 0.001; group × time: *p* = < 0.001. ( **D** ) Group effect: *p* = 0.053; time effect: *p* = < 0.001; group × time: *p* = < 0.001. *Within-group difference as a function of postoperative period; †between-group difference as a function of postoperative period. *p* -Values obtained using two-way repeated-measures analyses of variance (group: TP vs. RP or clamp: IR vs. SR + SC × time: pre-/postoperative period) followed by paired/independent samples *t* -tests following an interaction.

**Fig. 2:**
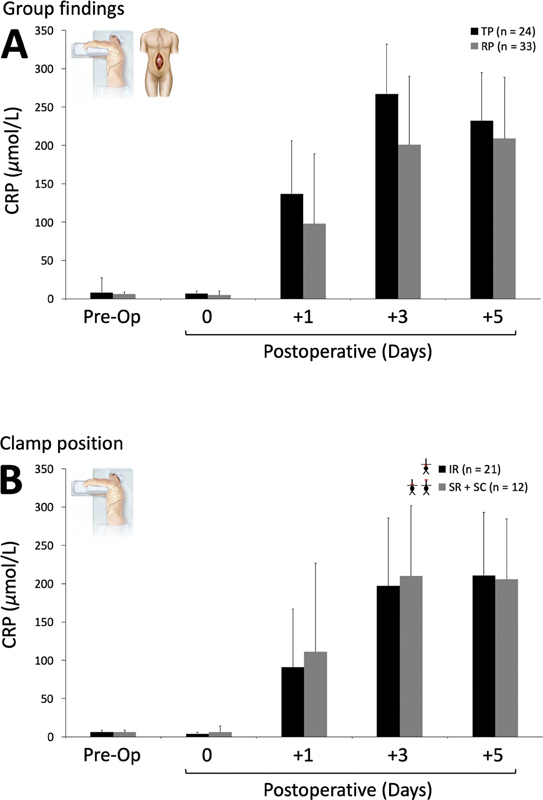
Systemic inflammation following transperitoneal (TP) and retroperitoneal (RP) abdominal aortic aneurysm repair. Values are mean ± standard deviation (SD). CRP, C-reactive protein. Group mean changes ( **A** ) further differentiated according to clamp position in RP patients ( **B** ). IR, infrarenal; SR, suprarenal; SC, supraceliac. Day 0 is the first sample taken on admission to critical care postoperatively. ( **A** ) TP + RP groups. Group effect: *p* = 0.002 (retrospective power of 0.895); time effect: *p* ≤ 0.001; group × time: *p* = 0.053 (greenhouse-Geisser correction). ( **B** ) RP group only. Group effect: *p* = 0.627; time effect: *p* ≤ 0.001; Group × time: *p* = 0.868 (greenhouse-Geisser correction). *p* -Values obtained using two-way repeated-measures analyses of variance (Group: TP vs. RP or clamp: IR vs. SR + SC × time: pre-/postoperative periods).

POMS scores were higher in TP patients owing to more pulmonary and gastrointestinal complications, translating into more time spent in hospital and HDU ( Table 3 ). Renal function was acutely impaired following surgery, confirmed by elevated serum creatinine and corresponding reduction in eGFR, before recovering to preoperative baseline levels, though the responses were not different between groups ( Fig. 1A, B ). Within the RP group, the renal impairment was more marked in patients receiving SR/SC compared with IR clamps ( Fig. 1C, D ). No patients from the TP or RP group required long-term renal replacement therapy after discharge from the hospital. Systemic inflammation inferred from changes in serum CRP was more marked in the TP compared with the RP approach, peaking on day 3 after surgery ( Fig. 2A ). No differences were observed according to clamp position in the RP group ( Fig. 2B ). There was no difference in hernias or bulges between the two groups ( Table 3 ).

**Table 3:** Postoperative morbidity and mortality

	Transperitoneal ( *n* = 24)		Retroperitoneal ( *n* = 33)		*p* -Value
*Morbidity:*					
POMS score [median (IQR)]	1.0 (1.0–2.75)		0.0 (0.0–0.5)		< 0.001
POMS score ≥ 1 [ *n* (%)]	19	(79)	8	(24)	< 0.001
*Complication:*					
Pulmonary [ *n* (%)]	6	(25)	1	(3)	0.034
Infection [ *n* (%)]	3	(13)	1	(3)	0.300
Renal [ *n* (%)]	5	(21)	4	(12)	0.470
Gastrointestinal [ *n* (%)]	15	(63)	0	(0)	< 0.001
Cardiovascular [ *n* (%)]	2	(8)	0	(0)	0.173
Neurological [ *n* (%)]	1	(4)	0	(0)	0.421
Hematological [ *n* (%)]	1	(4)	0	(0)	0.421
Wound [ *n* (%)]	1	(4)	1	(3)	1.000
Pain [ *n* (%)]	4	(17)	2	(6)	0.227
Hernias/Bulges [ *n* (%)]	2	(8)	3	(9)	1.000
*Length of stay:*					
Hospital days [median (IQR)]	10.5 (8.0–17.3)		7.0 (6.0–8.5)		0.001
HDU days [median (IQR)]	2.5 (2.0–3.0)		2.0 (2.0–2.0)		< 0.001
ICU days [median (IQR)]	0.0 (0.0–0.0)		0.0 (0.0–0.0)		0.558
*All-cause mortality:*					
30 d [ *n* (%)]	1	(4)	0	(0)	0.421
90 d [ *n* (%)]	1	(4)	0	(0)	0.421
2 y [ *n* (%)]	1	(4)	1	(3)	1.000
Survival days [median (IQR)]	1,507 (359–1,594)		1,597 (766–2,589)		0.345

Abbreviations: HDU, high dependency unit; ICU, intensive care unit; IQR, interquartile range; POMS, Postoperative Morbidity Survey (completed on day 5 after the operation).

Note: Values are median (IQR), frequency ( *n* /%), or mean ± standard deviation (SD). Abdominal aortic aneurysm (AAA) mortality classified within International Classification of Diseases, Tenth Revision (ICD-10) I71 “Aortic aneurysm and dissection.” *p* -Values obtained using independent samples *t* -tests, Chi-square tests, or Fisher's exact tests where cell counts were insufficient.

### Postoperative Mortality

One patient in the TP group died on day 5 after surgery due to an ischemic bowel. Mortality (30 days/90 days/2 years) was low and comparable across groups ( Table 3 ).

### Postoperative Costs

**Fig. 3:**
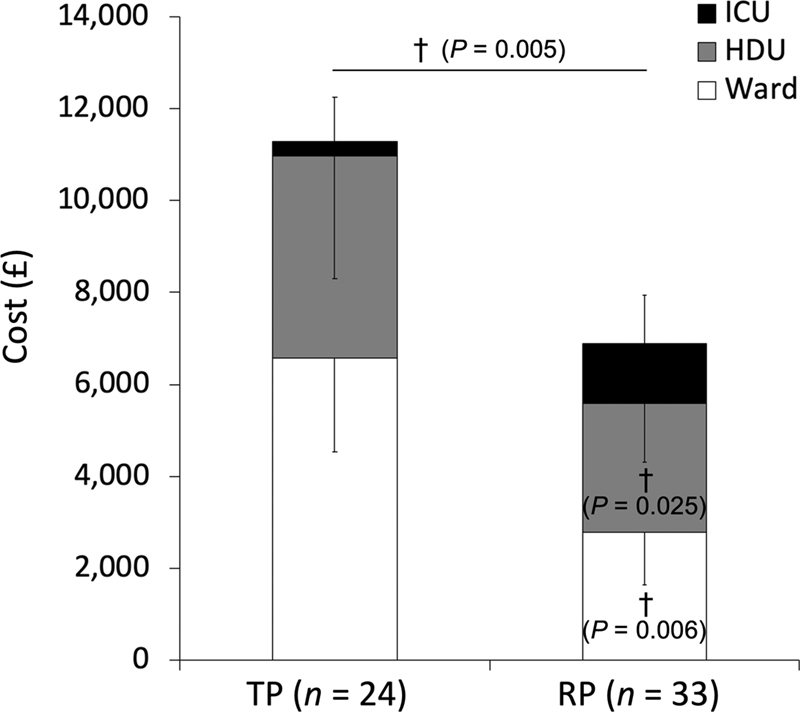
Hospital costs. Values are mean ± standard deviation (SD). HDU/ICU, intensive care/high dependency unit; TP, transperitoneal; RP, retroperitoneal. †Between-group difference. *p* -Values obtained using independent samples *t* -tests or Mann–Whitney *U* -tests.

Fig. 3 summarizes estimated hospital costs based on LoS and specific unit/ward costs. An RP patient cost on average 45% less compared with a TP patient, with a cost saving per patient of £5,080 (£6,209 ± 1,769 vs. £11, 289 ± 7,993) due predominantly to a reduced HDU and ward LoS.

## Discussion

This study has revealed two important findings. First, despite being at greater operative risk of renal complications subsequent to SR/SC cross-clamp placement, there were fewer postoperative pulmonary/gastrointestinal complications in patients undergoing the RP compared with the TP approach. Consequently, this translated into significantly less time recovering in hospital postoperatively, despite considerably more patients requiring a cross-clamp placed proximal to the renal arteries. Second, the RP approach was associated with less systemic inflammation reflected by consistently lower CRP levels that extended well into the postoperative recovery period. Importantly, these observations could not be attributed to differences in CRF or associated cardiovascular risk factors given that both groups of patients were, with the exception of sex, well matched. Collectively, these findings suggest that the physiological insult and corresponding postoperative morbidity associated with RP repair is lower compared with the more traditional TP approach despite a more anatomically complicated perirenal aorta.

These findings may seem counterintuitive given that anatomically, RP patients in this specific series pose a higher operative risk subject to a higher prevalence of SR/SC cross-clamps and increased potential for renal and mesenteric ischemia-reperfusion injury. Yet, despite this, they developed fewer pulmonary and gastrointestinal complications, which translated into faster recovery and hospital discharge. Other series have identified pulmonary complications as a leading risk factor prolonging LoS when performing open TP JRAAA repair. 24 25 Hence, this study supports offering the RP approach in patients with respiratory compromise including those with impaired CRF given that baseline respiratory function is an established independent predictor of postoperative pulmonary complications. 11

Possibly, the major advantage of the RP approach is the avoidance of entry into the peritoneal cavity and intestinal manipulation, procedures that are unavoidable consequences of a laparotomy. Abdominal entry and intestinal manipulation can compromise splanchnic perfusion and lead to mucosal barrier dysfunction, predisposing to postoperative complications. 26 In support of these observations, reduced intestinal permeability, endotoxemia, and interleukin (IL)-6 formation have been observed in patients undergoing RP compared with TP repair. 27 28

By focusing on the temporal kinetics of CRP release (an acute-phase reactant with a half-life of 19 hours, 29 synthesized and secreted by the liver and regulated almost exclusively by IL-6 30 ) our data further extend these findings and suggest that the operative trauma incurred during RP repair was less pronounced. The dedicated focus on CRP was predicated by its established sensitivity and specificity as an independent risk factor, 31 predictor, 32 and prognostic biomarker 33 in the setting of AAA disease and repair. However, future studies should also consider serial examination of additional (bloodborne) biomarkers including tumor necrosis factor-α and the ILs notably IL-1β, IL-8, IL-2, and IL-18 34 notwithstanding free radical biomarkers of oxidative-nitrosative stress 35 to compliment the current findings.

To date, only three randomized trials have compared RP and TP approaches for aortic surgery. 36 37 38 However, all trials focused exclusively on patients undergoing IR AAA repair and were further complicated by differences in baseline demographic and comorbid variables, without consideration of CRF, an albeit understandable exclusion given that these trials predated the current nearly routine use of CPET now in modern surgical practice. Furthermore, with surgical advances, a majority of patients in the modern era would now be considered for EVAR. Two of the studies showed no advantage of the RP over the TP approach with comparable LoS. 36 38 However, an albeit smaller study 37 confirmed shorter LoS and decreased ileus rates, yet the conclusion was not to recommend the RP approach as first line for IR AAA surgery.

Despite the limitations associated with the retrospective analysis of historical data, the present study, therefore, represents an important methodological advance, given that baseline demographics including cardiovascular risk factors, CRF, and medication were comparable across groups. Thus, from a design perspective, we were in a better position to isolate the impact of surgical technique on patient outcomes. The lower postoperative morbidity observed following RP repair cannot be accounted for by preoperative differences (i.e., elevation) in CRF, which other studies have linked with fewer postoperative complications. 10 11 12

Lastly, the shorter LoS associated with RP repair incurred significant cost savings. However, from a surgical perspective, the economic benefits need to be considered against the increasing numbers of patients who would be candidates for EVAR in the future, with associated shorter LoS compared with open repair. 39 The financial benefits provided by EVAR depend, in part, on the balance between the initial high cost of the stent graft and a cheaper, complication-free recovery time in hospital. 40 41 However, the development of endoleaks and/or increasing aortic sac diameters are recognized long-term complications after EVAR, possibly requiring further endovascular intervention after radiological surveillance. In the future, it is likely there will be patients anatomically unsuitable for conventional EVAR yet sufficiently fit following CPET to be considered for open repair.

## Conclusion

This study of patients turned down for uncomplicated EVAR but with equivalent comorbidities and CRF has identified that the RP approach to complex open AAA repair was associated with fewer postoperative pulmonary/gastrointestinal complications and lower systemic inflammation. This resulted in less time spent recovering in hospital compared with the more common TP approach. This was especially pertinent given the higher prevalence of proximal aortic clamp placement in RP patients indicating a higher postoperative risk of renal complications. Furthermore, the financial benefits highlighted by these findings justify the more routine use of the RP approach for patients undergoing complex AAA repair.
